# Exploring cellular behavior under transient gene expression and its impact on mAb productivity and Fc‐glycosylation

**DOI:** 10.1002/bit.26456

**Published:** 2017-11-16

**Authors:** Si N. Sou, Ken Lee, Kalpana Nayyar, Karen M. Polizzi, Christopher Sellick, Cleo Kontoravdi

**Affiliations:** ^1^ Department of Life Sciences Imperial College London London UK; ^2^ Centre for Synthetic Biology and Innovation Imperial College London London UK; ^3^ Centre for Process Systems Engineering, Department of Chemical Engineering Imperial College London London UK; ^4^ Cell Culture and Fermentation Sciences MedImmune Cambridge UK

**Keywords:** cell metabolism, galactosylation, glycosylation, monoclonal antibody, transient gene expression

## Abstract

Transient gene expression (TGE) is a methodology employed in bioprocessing for the fast provision of recombinant protein material. Mild hypothermia is often introduced to overcome the low yield typically achieved with TGE and improve specific protein productivity. It is therefore of interest to examine the impact of mild hypothermic temperatures on both the yield and quality of transiently expressed proteins and the relationship to changes in cellular processes and metabolism. In this study, we focus on the ability of a Chinese hamster ovary cell line to galactosylate a recombinant monoclonal antibody (mAb) product. Through experimentation and flux balance analysis, our results show that TGE in mild hypothermic conditions led to a 76% increase in q_P_ compared to TGE at 36.5°C in our system. This increase is accompanied by increased consumption of nutrients and amino acids, together with increased production of intracellular nucleotide sugar species, and higher rates of mAb galactosylation, despite a reduced rate of cell growth. The reduction in biomass accumulation allowed cells to redistribute their energy and resources toward mAb synthesis and Fc‐glycosylation. Interestingly, the higher capacity of cells to galactosylate the recombinant product in TGE at 32°C appears not to have been assisted by the upregulation of galactosyltransferases (GalTs), but by the increased expression of N‐acetylglucosaminyltransferase II (GnTII) in this cell line, which facilitated the production of bi‐antennary glycan structures for further processing.

## 1 INTRODUCTION

Transient gene expression (Wingens et al., 2015) offers an alternative to stable gene expression (SGE) for the rapid production of recombinant proteins required for discovery, early stage development, and pre‐clinical studies (Pham, Kamen, & Durocher, 2006). Previous studies have demonstrated that the introduction of mild hypothermic culture temperature in TGE systems can have a positive effect on recombinant protein yield (Galbraith, Tait, Racher, Birch, & James, 2006) by promoting high cell viability, high recombinant transcript expression, and increased mRNA stability (Cain et al., 2013; Marchant, Al‐Fageeh, Underhill, Racher, & Smales, 2008; Wulhfard et al., 2008), although the effect is expression vector‐dependent (Wulhfard et al., 2008). However, the relationship between cell metabolism, product synthesis, and post‐translational modification in the highly dynamic environment of TGE is currently not well described.

In this study, we compare the metabolic behavior, specific monoclonal antibody (mAb) productivity, and glycosylation in a Chinese hamster ovary (CHO) cell line undergoing TGE at physiological temperature (36.5°C) and with a shift to mild hypothermia (32°C). Two bioreactor experiments were carried out: one set of triplicate 14‐day fed‐batch cultures of the parental CHO cell line at 36.5°C transfected with IgG heavy chain (Starega‐Roslan et al., 2011) and light chain (LC) plasmid DNA 24 hr post inoculation and another set of triplicate cultures subjected to a temperature shift from 36.5 to 32°C 24 hr post‐transfection.

**Figure 1:**
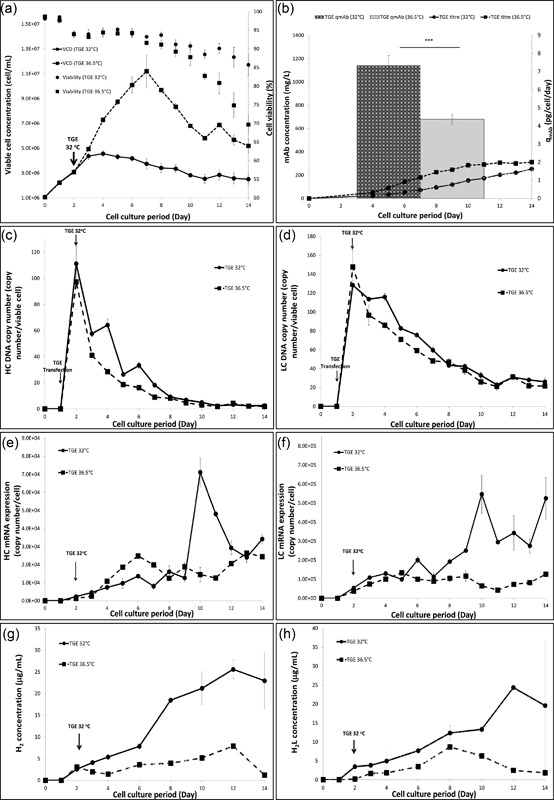
(a) Viable cell concentration and cell viability profiles and (b) q_mAb_ calculated based on final titre and IVCC, and mAb concentration profile for cultures grown at 36.5°C and with a temperature shift to 32°C. Concentration profiles of heavy (c) and light chain (d) DNA copy number, heavy (e) and light (f) chain mRNA, as well as H_2_ (g) and H_2_L (h) intracellular assembly intermediates of IgG molecules in TGE at 36.5°C and TGE at 32°C 24 hr post‐transfection. Results are average measurements (*n* = 3). Error bars represent standard deviation of measurements

Upon DNA transfection, there was a decrease in cell viability in response to the mild toxicity of the transfection reagent (polyethyleneimine, PEI) (Figure 1a). The introduction of mild hypothermia at early exponential phase in the second set of experiments further limited rapid cell division; cells enter a prolonged stationary phase with a reduced biomass production rate. This is a common outcome of mild hypothermia in which partial cell cycle arrest is often reported (Marchant et al., 2008). Despite the reduction in the integral of viable cell concentration (IVCC), cells at 32°C had prolonged viability, with 85% viability at harvest as opposed to 70% viability observed in cells cultured at 36.5°C. Figure 1b shows the volumetric and specific mAb productivity at these two temperatures. Despite a slightly higher volumetric yield observed in the TGE system at 36.5°C, average q_mAb_ was higher at 32°C in our system.

To investigate the difference in mAb productivity, we examined the DNA copy number, mRNA, and polypeptide expression levels at both temperatures. Figures 1c and 1d show that upon plasmid internalization on day 2, cells at 32°C exhibit slightly higher HC and LC DNA copy numbers than at 36.5°C until day 7. Figures 1e and 1f illustrate that both HC and LC mRNA copy numbers are higher during late stage culture at 32°C, with maximum mRNA levels observed on day 10. This is in agreement with the dynamic q_mAb_ profile where mAb production rate at 32°C is increased on day 10 (data not shown). In addition, overall increases in H_2_ (heavy chain dimer) and H_2_L (one light chain attached to heavy chain dimer) assembly intermediate concentrations were obtained when cells were cultured at 32°C (Figures 1g and 1h), with a significantly increased amount of H_2_L species beyond day 10, when q_mAb_ was also substantially higher. This suggests that the addition of LC does not appear to be limiting at 32°C in our cell line under these conditions.

**Figure 2:**
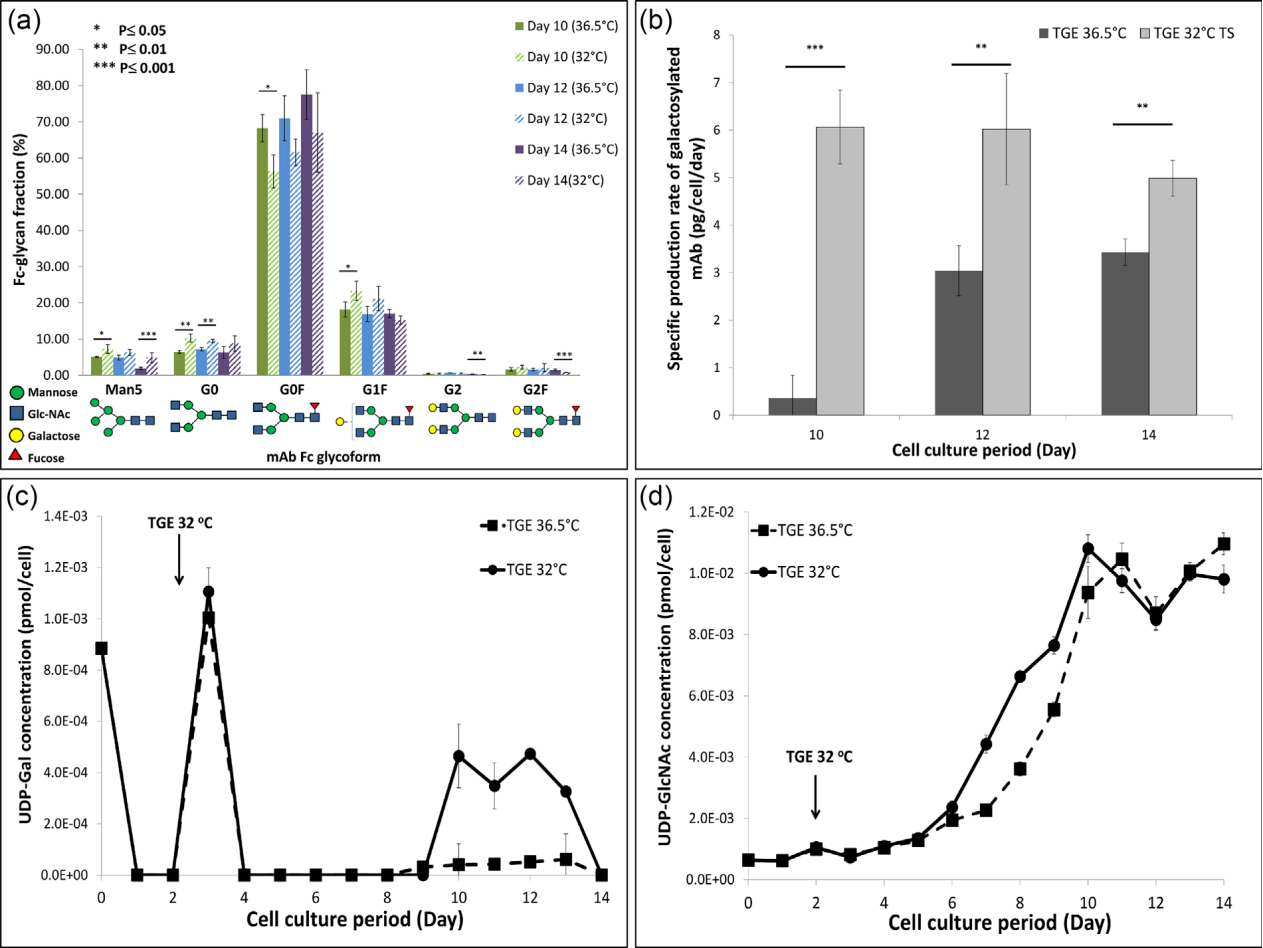
Glycan profile of the secreted IgG (a); specific production rate of galactosylated mAb (b); intracellular concentrations of UDP‐Gal (c) and UDP‐GlcNAc (d). Results are average measurements at 36.5°C (*n* = 3) and 32°C (*n* = 3). Error bars represent standard deviation. Statistical significance was calculated using a Student *t*‐test and was represented by: *p* ≤ 0.05(*), *p* ≤ 0.01(**), and *p* ≤ 0.001(***). TS, temperature shift

We further examined whether product glycosylation was affected by mild hypothermia by comparing the secreted mAb Fc‐glycan profiles at 36.5°C and at 32°C on days 10, 12, and 14. In contrast to the results observed in Sou et al. (2015) for SGE, the induction of mild hypothermia did not result in significant changes in the abundance of individual mAb glycan structures. The only exceptions were Man5 and G0 species, for which there was a slight increase in cultures conducted under mild hypothermic conditions (Figure 2a). However, when the specific production rate of galactosylated mAb (q_gal‐mab_) under each condition was considered, differences between TGE at 36.5°C and 32°C became more pronounced. Results in Figure 2b suggest that q_gal‐mab_ was significantly higher when this cell line was cultured at 32°C. Supplementary Table S1 shows increased values for the galactosylation index, that is mole of galactose added per mole of mAb produced, observed at 32°C.

From a metabolic perspective, the synthesis of nucleotide sugar donors (NSDs) determines the availability of building components for protein glycosylation. Figures 2c and 2d show the net intracellular concentrations of UDP‐Gal and UDP‐GlcNAc, respectively, for our system at the two temperatures. UDP‐Gal availability varied in late stage culture, with higher concentrations observed at 32°C. This variation, which follows closely the profile of UDP‐Glc (data not shown), may result from the higher specific glucose and lactate uptake rates observed in late stage culture at 32°C (supplementary Figure S1). The specific glucose consumption rate was approximately 60% higher at 32°C than in those at 36.5°C. In addition, approximately 74% of the lactate secreted during the exponential phase at 32°C was consumed at late stage culture compared to 12% at physiological temperature.

**Figure 3:**
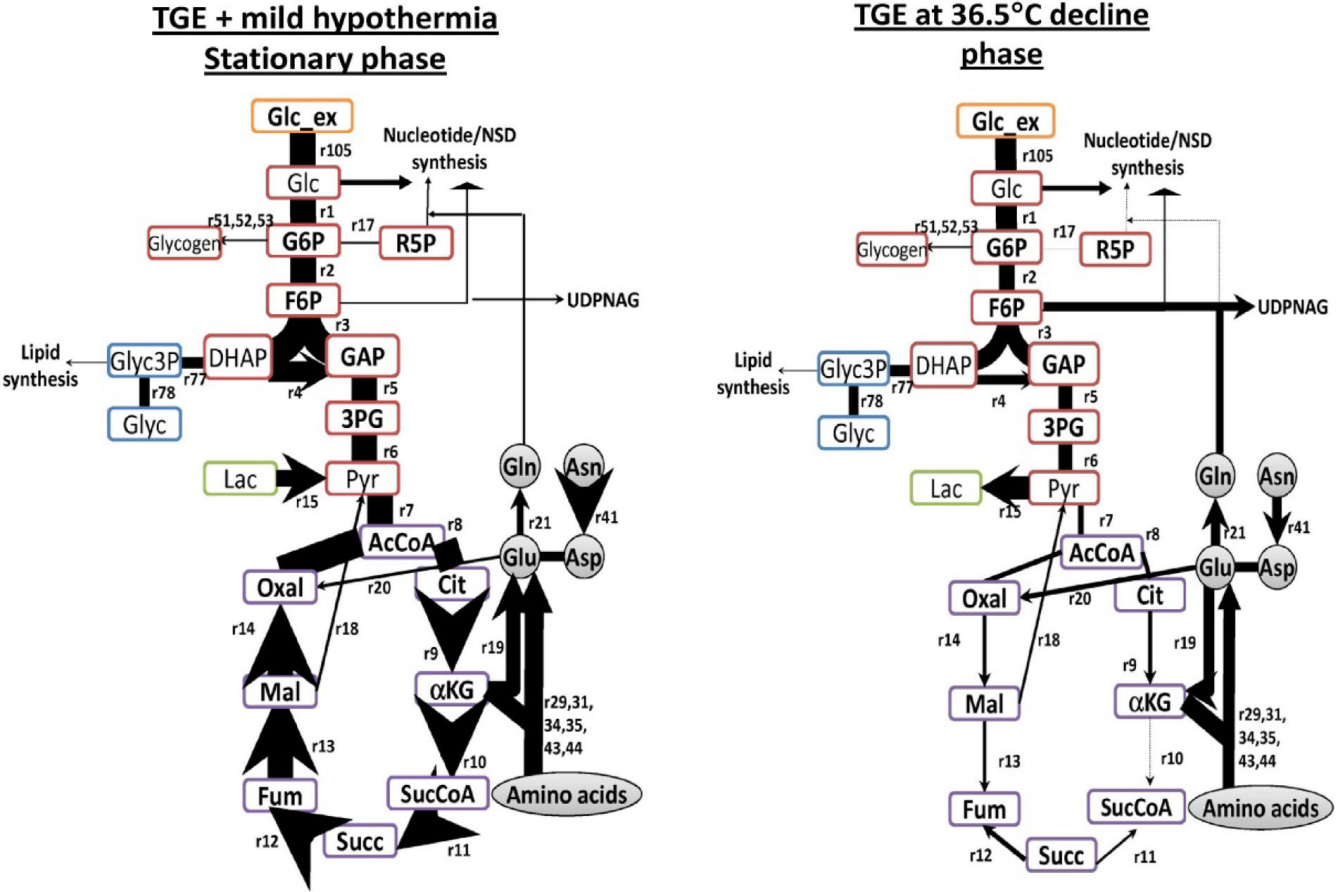
Central carbon metabolism of CHO cells at stationary phase in TGE at 32°C and decline phase at 36.5°C. Thickness of arrows indicates the relative magnitude of the carbon flux within the system. This figure is simplified to include carbon converted to glycerol, glycogen, and lactate production, as well as nucleotide, NSD, lipid, and key amino acid syntheses

From the experimental data, we calculated the consumption and production rates of each measured species. The rates calculated for the stationary phase at both temperatures (supplementary Table S2) show that at 36.5°C transient transfectants behave very differently from those at 32°C. To further elucidate the effect of temperature on product glycosylation in TGE, intracellular fluxes were calculated using flux balance analysis (FBA). The FBA was constrained with the calculated rates of exometabolites, maximum q_mAb_ and growth rate (μ). Despite not having an obvious stationary phase in the TGE system at 36.5°C, we analyzed carbon fluxes in the decline phase and compared them to those at the same time point for the 32°C system, which was still at stationary stage. The metabolic rates in supplementary Table S2 and the fluxome illustrated in Figure 3 show slower consumption of most amino acids, for example isoleucine, lysine, serine, phenylalanine, and tyrosine at 32°C, but increased flux toward mAb synthesis. There is also increased amino acid flux for higher energy and product generation during the stationary mAb‐producing phase at 32°C. Cells cultured at 32°C show higher TCA cycle fluxes when compared to those at 36.5°C, indicating a more efficient metabolic state. This is also evident from the flux of serine for pyruvate generation, which was calculated to be 10 times higher at 36.5°C, with most of it then converted into lactate as metabolic waste. In the case of NSD metabolism, the overall carbon and nitrogen fluxes going toward nucleotide, NSD and lipid syntheses are lower at 36.5°C (Figure 3).

**Figure 4:**
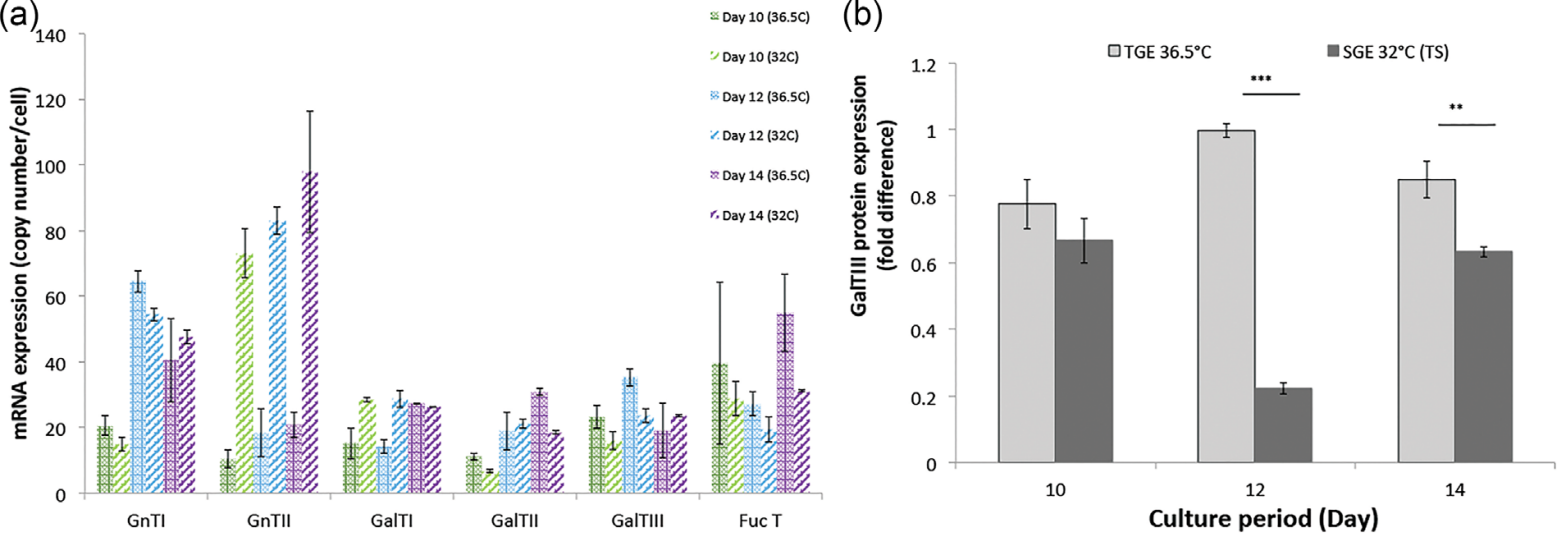
mRNA expression levels of six glycosyltransferases, namely GnTI, GnTII, GalTI, GalTII, GalTIII, and FucT (a) and relative difference in galactosyltransferase III (β‐GalTIII) protein expression (b). Results are average measurements at 36.5°C (*n* = 3) and 32°C (*n* = 3). Error bars represent standard deviation. Statistical significance was calculated using a Student *t*‐test and was represented by: *p* ≤ 0.05(*), *p* ≤ 0.01(**), and *p* ≤ 0.001(***)

In addition to the availability of NSD species, another factor that affects glycan processing is the expression of glycosyltransferases and NSD transporters involved in N‐linked glycosylation. Figure 4a shows that the gene expression levels of β‐GalT II (day 14) and III (day 12) and FucT (day 14) were significantly higher at 36.5°C than those at 32°C, suggesting that higher q_gal‐mAb_ was not due to an increase in galactosyltransferase expression in this case. Protein expression of β‐GalTIII was also higher at 36.5°C (Figure 4b). However, we also observed an increase in mRNA expression of GnTII under mild hypothermia. This suggests that the addition of UDP‐GlcNAc to form bi‐antennary glycan structures could, in fact, be the critical step that enables further mAb glycan processing at 32°C.

Our study demonstrates the impact of mild hypothermia on CHO cell metabolism and recombinant protein production in transiently transfected CHO cells. In addition to the beneficial effects of mild hypothermia on specific productivity of recombinant mAb that have been widely reported in both SGE and TGE systems (Codamo, Hou, Hughes, Gray, & Munro, 2011; Codamo, Munro, Hughes, Song, & Gray, 2011; Kantardjieff et al., 2010; Nam, Zhang, Ermonval, Linhardt, & Sharfstein, 2008; Wulhfard et al., 2008; Yoon, Kim, Song, & Lee, 2006; Yoon, Song, & Lee, 2003), we observed a re‐distribution of nutrients, metabolites, and energy from biomass formation toward recombinant protein synthesis and product glycosylation, in response to mild hypothermia. This resulted in not only increased q_mab_ but also increased fraction of galactosylated structures under mild hypothermia. The comparison of glycosyltransferase mRNA expression and protein levels between the two temperatures examined identifies GnTII as the driving force for enhanced specific rate of mAb galactosylation under mild hypothermia in our system by increasing the bi‐antennary glycoforms available for terminal N‐linked galactosylation. Although CHO cell lines in academic and industrial settings can vary greatly in their response to mild hypothermia and TGE due to their different origin, adaptation to different media and cultivation conditions, this study highlights the importance of analyzing experimental data on a per cell basis in order to correctly identify intracellular bottlenecks to achieving the desired product quality.

## 2 MATERIALS AND METHODS

### 2.1 Cell line and bioreactor experiments

A CHO transient host cell line (CHO‐T) (MedImmune, Cambridge, UK) was revived and grown in CD‐CHO medium (Life Technologies, Paisley, UK) at 36.5°C, in a 5% CO_2_ humidified incubator. Viable cell density and cell viability were quantified by ViCell® (Beckman Coulter, CA). Experiments were carried out in triplicate 1.5 L stirred tank DASGIP bioreactors (DASGIP Technology, Juelich, Germany) at pH 6.9 ± 0.1 with an initial culture volume of 0.9 L and initial viable cell density of 8 × 10^5^ cells/ml. The temperature was controlled at 36.5°C and was shifted to 32°C for one set of triplicates 24 hr post transfection. On days 2, 4, 6, 8, 10, and 12 the reactors were supplemented with 10% v/v CD EfficientFeed™ C AGT™ Nutrient supplement (Life Technologies). Foaming was managed by 5 ml additions of 15% antifoam C (Sigma–Aldrich, Dorset, U.K.). Transfection was carried out 24 hr after cell inoculation, at a cell density of 2 × 10^6^ cells/ml following the protocol described in Daramola et al. (2014). Heavy and light chain vectors (MedImmune, Cambridge, U.K.) were co‐transfected using linear PEI 25,000 Da (Polysciences Europe GmbH, Germany) at a PEI:DNA ratio of 5:1 and diluted in 150 mM NaCl, with 1 mg total DNA per 1 L of culture volume.

### 2.2 Analytical assays

The total DNA and RNA content was extracted from cell pellets containing 5 × 10^6^ cells according to the manufacturer's protocols for the All prep DNA/RNA mini purification kit (Qiagen, Manchester, U.K.). A 300 ng of extracted total RNA from each sample was reversed transcribed into cDNA using the QuantiTect Reverse Transcription Kit (Qiagen) and 1 μl of the RT Primer Mix according to the manufacturer's instructions. To determine the DNA copy number and mRNA expression levels of mAb heavy (HC HuG1) and light chains (LC HuKappa) in each sample, polymerase chain reaction (PCR) and quantitative real‐time PCR (qRT‐PCR) were performed, respectively. Each DNA or cDNA sample was analyzed using triplicate PCR experiments in a 96 well‐plate using 5 μl of 2× SYBR Green Supermix (Sigma–Aldrich), 0.64 μl of DNA (or cDNA) and 500 nM of each primer pair and ddH_2_O to make a total of 10 μl of reaction volume per well. Experiments were initiated with SYBR Green activation at 95°C for 3 min followed by 40 cycles of 95°C for 30 s, 60°C for 75 s, and 72°C for 30 s. DNA melting curve was performed from 65 to 95°C (read every 0.3°C) to verify product integrity. mRNA results were compared to the C_t_‐number of house‐keeping gene β‐actin for relative analysis, while the gene copy number was calculated from a calibration curve of known DNA concentrations. Primer sequences of glycosyltransferases are available on request.

Intracellular mAb polypeptides, assembly intermediates, and galactosyltransferase III (GalTIII) protein expression analyses were performed as discussed in Sou et al. (2015). Intracellular nucleotides and NSDs were successfully extracted using an acetonitrile extraction method based on Dietmair, Timmins, Gray, Nielsen, and Kroemer (2010). Viant, Bundy, Pincetich, de Ropp, and Tjeerdema (2005). HPAEC analysis was conducted based on the method presented in del Val, Kyriakopoulos, Polizzi, and Kontoravdi (2013) using a CarboPac PA‐1 column with a PA‐1 guard column (Dionex, CA). Samples were eluted using a gradient of E1 (3 mM NaOH) and E2 (1.5 mM sodium acetate in 3 mM NaOH) buffers as mobile phases. All species were detected at two absorbance wavelengths: 271.6 nm for all cysteine‐bearing species and 262.1 nm for all other compounds. Extracellular concentrations of ammonia, lactate, and glucose were quantified using the YSI Bioprofiler 800 (Nova Biomedicals, MA). Extracellular amino acid concentrations were determined using a Waters Acquity ultra‐performance liquid chromatography (UPLC, Walters, Hertfordshire, U.K.) with the AccQ‐tag kit used according to the manufacturer's instructions. A Protein‐A affinity chromatography method developed by MedImmune, Cambridge, U.K. was performed to analyze secreted mAb concentration.

### 2.3 mAb glycan analysis

mAb samples from crude supernatants were purified to a concentration between 1.25 and 7.5 mg/ml prior to glycan analysis using the ProfilerPro Glycan Profiling Kit (PerkinElmer, MA), which was conducted following the method described in Sou et al. (2015).

### 2.4 Flux balance analysis (FBA)

FBA was performed in the R workspace (R Development Core Team, 2010) with the Sybil package (Gelius‐Dietrich, Desouki, Fritzemeier, & Lercher, 2013). Fluxes were estimated using the metabolic network constructed by Kyriakopoulos and Kontoravdi (2014) based on the network proposed by Carinhas et al. (2013), while the biomass composition used was that proposed by Selvarasu et al. (2012) for CHO cells. The model was optimized by assuming maximum IgG formation at stationary phases in the TGE system. FBA calculations were constrained by experimentally quantified concentrations of extracellular amino acids, glucose, lactate, ammonia, secreted IgG, and viable cell density. One standard deviation was used as the upper and lower limits while the remaining extracellular fluxes were set at ±20% (Carinhas et al., 2013). The model was extended to incorporate fluxes for lipid synthesis by including choline. GlcNAc, GalNAc, Man, Fuc, Gal, and Neu5Gc were added into the biomass equation to examine the glucose fluxes to nucleotide and NSD production for glycosylation. The amount of each NSD necessary for host cell protein glycosylation was estimated based on the results from the MS glycan study in Stanley (2010), as well as the occurring frequency of N‐ and O‐linked glycans based on Apweiler, Hermjakob, and Sharon (1999). These data were then used to calculate the respective stoichiometric coefficients that were incorporated into the biomass equation and used in the FBA analysis.

## Supporting information

Additional Supporting Information may be found online in the supporting information tab for this article.

**Figure S1**. Specific glucose consumption rate (left) and extracellular lactate concentration profile (right) for cultures grown at 36.5°C and with a temperature shift to 32°C under TGE.
**Table S1**. Galactosylation index of secreted mAb (mole of galactose per mole of mAb), under TGE at 36.5°C and with a temperature shift at 32°C.
**Table S2**. Average specific metabolic production and consumption rates for TGE at 36.5°C and with a temperature shift to 32°C.
