# Supplementary material for: Exploring cellular behavior under transient gene expression and its impact on mAb productivity and Fc‐glycosylation
Source: Biotechnol Bioeng. 2017 Nov 16;115(2):512–8. doi: 10.1002/bit.26456 (PMC5765507; doi:10.1002/bit.26456)
Supplement: Supplementary file 1 — Figure S1. Specific glucose consumption rate (left) and extracellular lactate concentration profile (right) for cultures grown at 36.5°C and with a temperature shift to 32°C under TGE. Table S1. Galactosylation index of secreted mAb (mole of galactose per mole of mAb), under TGE at 36.5°C and with a temperature shift at 32°C. Table S2. Average specific metabolic production and consumption rates for TGE at 36.5°C and with a temperature shift to 32°C. [file BIT-115-512-s001.docx]

# Supporting Information

# Exploring cellular behaviour under transient gene expression and its impact on mAb productivity and Fc-glycosylation

Si Nga Sou^1,2,3^, Karen M. Polizzi^1,2^, Ken Lee^4^, Kalpana Nayyar^4^,

Christopher Sellick^4#^, Cleo Kontoravdi^3# *^

^1^Department of Life Sciences, Imperial College London, London SW7 2AZ, U.K.

^2^Centre for Synthetic Biology and Innovation, Imperial College London, London SW7 2AZ, U.K.

^3^Centre for Process Systems Engineering, Department of Chemical Engineering, Imperial College London, London SW7 2AZ, U.K.

^4^Cell Culture and Fermentation Sciences, MedImmune, Granta Park, Cambridge, CB21 6GH, U.K.

# These authors are joint last author

* To whom correspondence should be addressed

Dr Cleo Kontoravdi

Imperial College London

Department of Chemical Engineering

ACEX 516

South Kensington, London

SW7 2AZ, UK

Tel: +44 (0)20 7594 6655, Fax: +44 (0)20 7594 6606

Email: [cleo.kontoravdi@imperial.ac.uk](mailto:k.polizzi@imperial.ac.uk)

**
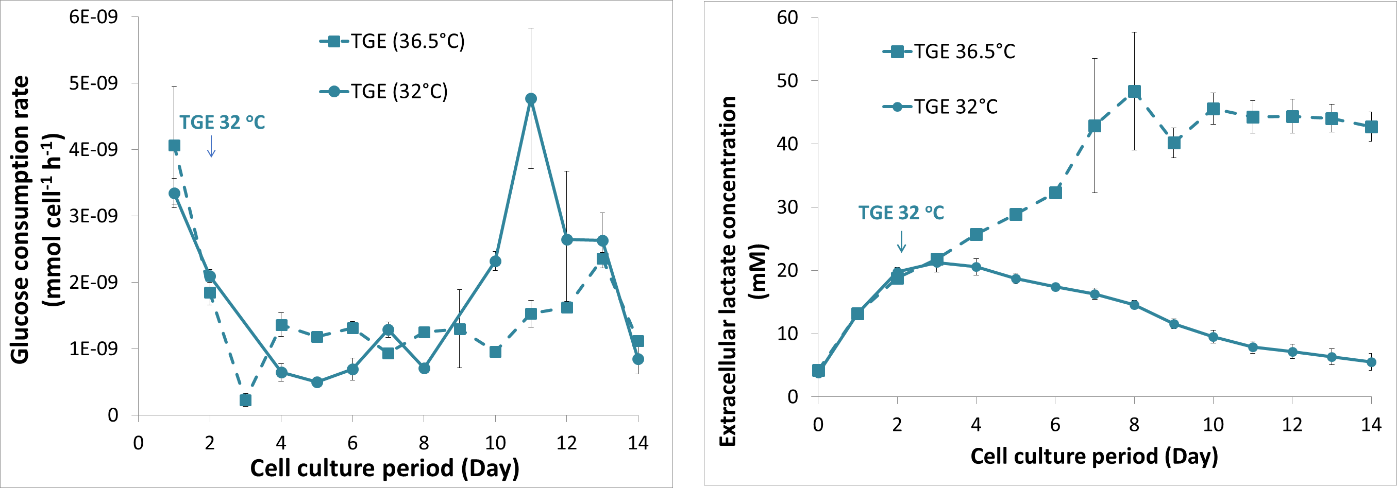
**

**Figure S1.** Specific glucose consumption rate (left) and extracellular lactate concentration profile (right) for cultures grown at 36.5°C and with a temperature shift to 32°C under TGE. Results are average measurements (n=3) and error bars represent standard deviation.

**Table S1.** Galactosylation index of secreted mAb (mole of galactose per mole of mAb), under TGE at 36.5°C and with a temperature shift at 32°C. Results are averaged measurements at 36.5°C (n=3) and 32°C (n=3).

| **Galactosylation index (mole of galactose per mole of mAb)** | | | | |
| --- | --- | --- | --- | --- |
| **Day** | **TGE 36.5^o^C** | | **TGE 32^o^C TS** | |
| 10 | 0.45 | ± 0.02 | 0.58 | ± 0.03 |
| 12 | 0.43 | ± 0.03 | 0.53 | ± 0.04 |
| 14 | 0.41 | ± 0.02 | 0.42 | ± 0.01 |

**Table S2.** Average specific metabolic production and consumption rates for TGE at 36.5°C and with a temperature shift to 32°C. Results are averaged measurements at 36.5°C (n=3) and 32°C (n=3).

|  | **Consumption/production rate (femtomol/cell/day)** | |
| --- | --- | --- |
|  |  | |
|  | **36.5°C** | **32°C** |
|  | **TGE** | |
| **Ala** | 106.8 | 152.0 |
| **Amm** | 18.0 | 38.8 |
| **Arg** | -13.5 | 5.0 |
| **Asn** | -118.0 | -308.0 |
| **Asp** | -80.5 | 159.2 |
| **Glc** | -346.5 | -480.5 |
| **Gln** | 47.5 | 64.8 |
| **Glu** | -9.4 | 2.4 |
| **Gly** | 87.4 | 72.4 |
| **His** | -0.5 | 18.7 |
| **Ile** | -27.4 | -1.9 |
| **Lac** | 481.4 | -262.7 |
| **Leu** | -94.7 | -97.5 |
| **Lys** | -23.6 | -1.8 |
| **Met** | -12.1 | -14.2 |
| **Phe** | -14.2 | -7.6 |
| **Pro** | -25.6 | -27.8 |
| **Ser** | -156.7 | -123.5 |
| **Thr** | -16.8 | 2.8 |
| **Trp** | -5.0 | -1.6 |
| **Tyr** | -9.1 | 6.1 |
| **Val** | -56.3 | -51.4 |
| **Negative values indicate consumption and positive values indicate production.** | | |
